# Tolerance of roadside and old field populations of common teasel (*Dipsacus fullonum* subsp. *sylvestris*) to salt and low osmotic potentials during germination

**DOI:** 10.1093/aobpla/plt001

**Published:** 2013-01-09

**Authors:** Laura L. Beaton, Susan A. Dudley

**Affiliations:** 1 Department of Biology, Life Science Building, McMaster University, 1280 Main Street West, Hamilton, Ontario, L8S 4K1, Canada; 2 Science Department, Royal Botanical Gardens, PO Box 339, Hamilton, Ontario, L8N 4H4, Canada; 3 Present address: Department of Biology, York College, City University of New York, 94-20 Guy R. Brewer Blvd, Jamaica, NY 11451, USA

**Keywords:** *Dipsacus fullonum*, drought tolerance, old-fields, roadsides, salt tolerance.

## Abstract

Plants inhabiting degraded habitats must contend with stressful environments. However, their ability to adapt may be constrained by available genetic variation and genetic correlations between traits. Here, we examine the correlation between salt and drought tolerance in germinating seeds from contrasting populations of common teasel (*Dipsacus fullonum* subsp. *sylvestris*) growing on roadsides that experience high salinity due to de-icing salts, or growing in an old field site, remote from roadsides and free of salinity stress. We examined the contribution of drought and salinity tolerance to the tolerance of roadside conditions in seedlings from five maternal families from three roadside and three old field populations. Germination and early growth were compared under high salinity, low water potential set at −0.5 MPa with solutions of polyethylene glycol 8000, sodium chloride or vermiculite wetted to −0.5 MPa with distilled water. Root length and the emergence of cotyledons (where appropriate) were used as a measure of performance. Maternal families from roadside populations displayed greater tolerance of both high salinity and drought than families from old field populations. However, no maternal family possessed tolerance to both drought and salinity. Salt and drought tolerance during germination were not correlated, indicating that they are separate traits in this species.

## Abstract (precis)

Maternal families from roadside populations of *Dipsacus fullonum* subsp. *sylvestris* displayed greater tolerance of both high salinity and drought during germination than families from old field populations. However, the tolerances were not correlated, indicating that they are separate traits.

## Introduction

Populations inhabiting environments created by human activities must successfully contend with multiple strong selection pressures, often simultaneously. Their ability to respond is constrained both by the available genetic variation and by genetic correlations between tolerance traits (Caruso 2004; Etterson 2004). If adaptive traits are negatively associated, then tolerance to one stressor must come at a cost of tolerance to the other. However, in some instances, traits may be overlapping, so that tolerance towards one stress confers tolerance towards a second (Cullium *et al*. 2001). Here, we ask whether salt tolerance observed in roadside populations of the salt-sensitive common teasel (*Dipsacus fullonum* L. subsp. *sylvestris*) is a reflection of the variation in drought tolerance, a selection pressure more likely to have been encountered by this glycophytic species prior to the imposition of salinity stress from modern highway de-icing.

Salt and drought tolerance are often assumed to be related. Their intricate relationship has been demonstrated in a microarray analysis of *Arabidopsis thaliana*. In this model species, 141 of the 194 genes activated by salt stress were also activated by drought stress, and 48 of the 89 genes down-regulated by salt stress were also down-regulated by drought (Seki *et al*. 2002). High salinity lowers the osmotic potential of the soil, thereby inhibiting water uptake. Therefore, salt stress and drought stress are often considered as overlapping traits. Consequently, plants that are highly drought tolerant are often expected also to have enhanced tolerance to salinity. However, high salinity also exposes plants to elevated levels of sodium (Na^+^) and chloride ions, which have directly harmful effects. Although the exact mechanism of Na^+^ toxicity remains undiscovered, Na^+^ is known to compete with potassium (K^+^) for membrane binding sites (Epstein 1998). When exposed to high salinity, most plants sequester excess Na^+^ in leaf vacuoles. This helps to protect their cellular functions, particularly those involving K^+^. However, when amounts of Na^+^ exceed vacuolar storage capacity, Na^+^ ions accumulate in the cytoplasm. Cells will then either die from toxic effects or begin to pump Na^+^ out to the apoplast, where it accumulates in the cell walls. The resulting reduction in water potential in the apoplasm causes cells to dehydrate and eventually to die (Munns 1993, 2002). Thus, high salinity has more detrimental and varied effects than drought stress alone.

Both natural and agricultural species exhibit considerable variation in salt tolerance (Ashraf 1994; Hester *et al*. 1996; Glenn *et al*. 1999). Several examples of a rapid evolution of salt tolerance in newly created saline habitats indicate that high salinity imposes strong selection pressures for salt tolerance. In temperate regions, the application of de-icing salts, primarily NaCl, has resulted in the formation of highly saline soils along roadsides (Westing 1969; Davison 1971; Thompson and Rutter 1986; Thompson *et al*. 1986*a*, *b*; Labadia and Buttle 1996). Recently, it has been shown that roadside populations of *D. fullonum* are more tolerant of high soil salinity during early seedling growth than populations located in more benign environments (i.e. the old fields) (Beaton and Dudley 2004, 2007). However, it remains unclear whether drought tolerance may pre-adapt salt-sensitive species (glycophytes) such as *D. fullonum* to high salinity. In this study, we examine the role that drought tolerance plays in conferring the observed tolerance towards high salinity during germination and early seedling growth.

Unlike plants inhabiting many naturally saline environments, plants inhabiting roadsides experience the highest salt levels during germination and seedling establishment because salt levels are highest in the spring following the winter application of de-icing salts. In addition, seeds and seedlings inhabit the top 2 cm of soil, which will experience the greatest fluctuations in both salinity and moisture level. Because salt levels decline slowly throughout the summer as ions are leached out of the soil by rain (L. L. Beaton, unpubl. data) and because adult plants have a greater rooting depth, the selection pressures for salt and drought tolerance will be greater during the critical juvenile stages of life. Although seedling salt and drought tolerance may not reflect tolerance in the adult plant, plants that cannot tolerate high salinity during germination and establishment will not survive in the harsh, springtime roadside environment. For this reason, we focused on salt and drought tolerance during germination and establishment. Not only are the juvenile stages of life critical to the fitness of plant populations, maternal effects add an additional level of complexity to traits expressed during germination and an additional mechanism by which salt tolerance may be conferred (Beaton and Dudley 2007).

Investigating the role of low water potential in salt toxicity is methodologically challenging. Compounds such as mannitol have been used to impose osmotic stress on plants for comparison of responses to high salinity. However, these low-molecular-weight substances can be taken up by the plant, and may then either have toxic effects or be used by the plant for osmotic adjustment. It is impossible to differentiate the effects of the low water potential from the effects of the solute itself. Polyethylene glycol (PEG) has the advantage that it is not taken up by plant cells. Although at one time PEG was thought to be the ideal substance for studies of water stress because it was believed not to have any toxic properties, further research has shown that PEG has deleterious impacts on plants that are unrelated to low water potentials. For example, PEG solutions are extremely viscous and create low-oxygen environments for the roots by impeding oxygen influx into the respiring roots (Mexal *et al*. 1975). Hypoxic environments are extremely stressful for plants and cause large reductions in root growth (Drew 1997). An alternative to PEG or other various sugars was developed by Sharp *et al*. (1988). This takes advantage of the matric potential of vermiculite, rather than the osmotic potential of solutes. Through the careful addition of water, vermiculite can be adjusted to impose the same water potential as a comparable salt solution. Although this appears to be an ideal solution to the methodological problem, vermiculite not only lowers matric potential but also adds mechanical resistance to the growing medium, i.e. it gives the roots an opposing resistance that can alter morphology. However, because examination of stress tolerance focuses on the difference in response of genotypes to stress compared with favourable conditions rather than their growth in any one condition, this limitation can be overcome by comparing root length in the control versus drought treatments. It is important that the vermiculite is not packed so firmly that it creates a distorting mechanical impedance. Taking this precaution ensures that any differences in root length between the genotypes compared with the controls are not confounded by mechanical pressures. For these reasons and for completeness, we utilized both vermiculite and PEG to impose low water potentials.

Seeds of *D. fullonum* were collected from five field-pollinated maternal families from each of three old field populations and three roadside populations. Because *D. fullonum* is an obligately outcrossing species (Werner 1975), we assume that all the seeds from one maternal plant are at least half siblings. The use of field-collected seeds adds complexity to the study, as seedling performance reflects a combination of maternal environment effects as well as maternal effects and genetic traits. However, responses of the germinating seeds to the treatments potentially better reflect their performance under field conditions than would the seeds of plants grown for one generation in a common garden. Such maternal environmental effects can be adaptive (Lacey and Herr 2005). Although we are not able to distinguish genetic traits from traits derived from maternal effects, ignoring the potential important role of adaptive maternal environmental effects by relying on seed produced from plants grown in a common garden could significantly underestimate the salt and drought tolerance present in this species.

We germinated *D. fullonum* seeds collected from five field-pollinated maternal families from each of three old field populations and three roadside populations in either a control or one of three low-water-potential treatments of −0.5 MPa imposed using salt (NaCl), PEG or vermiculite. We used root length after 10 days and the emergence of cotyledons as measures of performance. We asked the following questions: (i) Do roadside and old field populations differ in their tolerance to high salinity and low osmotic potentials? (ii) Is there variation for growth in low water potentials within and among the populations? (iii) Are salt tolerance and tolerance of low osmotic potentials correlated traits?

## Methods

### Species description

*Dipsacus fullonum* subsp. *sylvestris* (common teasel), also referred to as *D. sylvestris*, is a native of Europe and was first recorded in Canada in 1877. It is found primarily in old fields (i.e. abandoned farmers' fields) and roadsides. *Dipsacus fullonum* is a monocarpic perennial, which grows vegetatively as a rosette for at least 1 year. Rosettes may reach 60 cm in diameter. In the spring, if the plant has reached a critical size, it bolts to form an upright flowering stem (Werner 1975) that reaches 0.5–2.5 m by July. Flowering occurs from July to September and seeds are shed throughout the fall. A single plant can produce up to 3000 seeds (Werner 1975).

### Seed collection

Six *D. fullonum* populations were chosen for study: three were located immediately adjacent to Highway 403 between Hamilton and Burlington, Ontario, Canada, and three were located in old fields, ∼3 km away from Highway 403. Highway 403 is a six-lane highway carrying 45 000–75 000 automobiles each day (Ontario Ministry of Transport 1994). Highway populations were located in full sunlight, 2–10 m from the road's edge. At all the roadside sites, the surrounding vegetation was primarily grasses and/or *Coronilla varia* planted following highway construction to stabilize the soil, together with some typical old field species (e.g. *Solidago* sp., *Daucus carota*). All three roadside sites had high Na^+^ levels in the spring, when roadside salinity is at its peak, although the amount of Na^+^ in the soil was highly variable. In March, the soil from the location of roadside population 3 had substantially higher Na^+^ levels (5.49 ± 2.1 mg g^–1^) than that from the locations of roadside populations 1 (0.88 ± 0.03 mg g^–1^) and 2 (0.6 ± 0.08 mg g^–1^). The location of roadside population 1 was farther from the highway than the other two collection sites, which may have contributed to the lower Na^+^ levels. Roadside population 2 had a gravely, silty soil, unlike the other two collection sites, where the soil was a dense clay. The roadside population 1 site was very close to the elevation of the highway with no intervening ditch. The three populations were several kilometres apart on the highway. It is unclear whether gene flow occurs between the populations. Although *D. fullonum* seeds do not have an obvious dispersal mechanism, they do float (L. L. Beaton, pers. observ.) and may be swept through roadside ditches in heavy rain or during snow melts. Gene flow between populations may also be achieved through pollen dispersal since this species is frequently visited by a variety of different insect pollinators, including butterflies (L. L. Beaton, pers. observ.), which have been shown in other areas to make frequent use of roadside corridors (Saarinen *et al*. 2005).

Old field populations were located on conservation lands of the Royal Botanical Gardens, Hamilton, Ontario, Canada, and were growing in full sunlight. The surrounding vegetation was dominated by grasses with a wide variety of forbs. The three old field populations were of similar size, ∼25–40 plants. They were all located near the Niagara escarpment with one population located at the top of the escarpment, and two located at lower elevations. Measurements of Na^+^ levels in soil at the sites were 0 mg g^–1^. Seeds were collected from 15–20 randomly selected plants from each population. Following collection, seeds were stored in paper envelopes at room temperature. Seeds from each plant were stored separately. All the seeds from one plant are referred to as a maternal family. Five maternal families from each population were randomly selected for study.

### Treatments

Treatments for seedling growth conditions were distilled water (control) or one of three low-water-potential treatments of −0.5 MPa: salt (NaCl), PEG (PEG 8000) or vermiculite (rinsed, medium grade ‘Therm o rock’ mixed with distilled water). Previous trials (Beaton and Dudley 2004) demonstrated that at this salinity level, seedlings were harmed by stress, but were still able to grow roots. The water potentials of the three treatments were measured using a Wescor HR-33T Dew Point Microvoltometer (Wescor, Logan, UT, USA). The water potentials of the PEG and salt treatments were calibrated by soaking small pieces of filter paper in the solutions. Small pieces of vermiculite were selected randomly for measurement. Two successive pieces of vermiculite within the appropriate range of water potential were deemed a satisfactory measure of the water potential of the vermiculite. All containers containing treatment materials were sealed with Parafilm until the time of the experiment (<24 h).

### Experimental design

For each of the four treatments, 10 seeds from each maternal family from each population from each site were placed in 90-mm plastic Petri dishes containing either 5 mL of water, salt solution or PEG solution, or 15 mL of vermiculite. The trays containing vermiculite were filled to a depth of ∼3–5 mm. Seeds were not covered by vermiculite, but were exposed to the same light levels as seeds in the solution treatments. Each family/treatment combination was replicated twice, for a total of 20 seeds in two Petri dishes per family per treatment. Petri dishes were lined with Whatman qualitative filter paper (except those trays receiving the vermiculite treatment) and sealed with Parafilm to prevent evaporation (Almansouri *et al.* 2001; Cornaglia *et al*. 2005). Seeds were germinated under a mixture of natural and artificial light at room temperature. After 10 days, root length was measured and the emergence of cotyledons was recorded.

### Tolerance

The salt, PEG and vermiculite tolerance of each maternal family was determined by dividing the average length of the main root axis of the maternal family in the salt, PEG or vermiculite treatment by the average length of the main root axis in the control treatment (Beaton and Dudley 2004, 2007). Salt tolerance of each family was also assessed by dividing the number of seedlings with emerged cotyledons per family in the salt treatment by the number of seedlings with emerged cotyledons per family in the control treatment. Drought tolerance in vermiculite and PEG solutions could not be assessed in this way because very few seeds had emerged cotyledons in the vermiculite treatment and no seedlings had emerged cotyledons in the PEG treatment.

### Statistical analysis

Statistical analysis was conducted using SAS version 8.2 for Windows. Root length was log transformed prior to analysis to normalize the data. A nested mixed-model analysis of variance (ANOVA) (PROC MIXED) was conducted to examine the effect of the treatments on the root length of the germinating seedlings. Pre-planned contrasts between treatments were examined using the pdiff option of the LSMEANS statement. Seeds that did not germinate were not included in the analysis. Tolerance data were reciprocally transformed to correct for the heteroscedasticity of the data. The correlation between the average salt, PEG and vermiculite tolerance of maternal families from each site (the roadside and the old field) was determined [PROC CORR (Pearson correlation)]. In addition, the correlation between salt, PEG and vermiculite tolerance as indicated by root length, and the salt tolerance as indicated by cotyledon emergence was determined (PROC CORR). A series of *F*-tests (PROC GLM) were employed to examine the differences in tolerance between the sites (old field and roadside).

The genetic correlations between the root growth in the control, salt, PEG and vermiculite treatments of the maternal families from each site (the roadside and the old field) were calculated following (Astles *et al*. 2006):
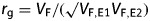



where *V*_F_ is the variance component due to overall family effects
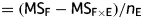



*V*_F,E*i*_ is the variance component of families in each treatment
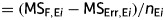



The mean square values of the overall family variance components (*V*_F_) were obtained by conducting mixed-model ANOVAs (PROC GLM with a RANDOM statement) examining the impact of the family (random factor), the treatment (fixed factor) and their interaction (random factor) on the log root length for each pair of treatments separately. The mean square values of the variance components for the family in each treatment were obtained by conducting mixed-model ANOVAs (PROC GLM with a RANDOM statement) examining the impact of the family (random factor) on the log root length for each treatment separately. The significance of genetic correlations between root growth in the control, salt, vermiculite and PEG treatments was estimated from family mean correlations (PROC CORR).

An ANOVA (PROC GLM) was conducted to examine the effect of the treatments and the collection site on the average percentage of seedlings with emerged cotyledons of the maternal families. Pre-planned contrasts between sites and treatments were examined using the pdiff option of the LSMEANS statement.

## Results

### Germination

Germination levels were significantly different between the treatments [*F* = 16.08; *P* = 0.0002 (PROC MIXED)] with fewer seeds (mean = 7.4 ± 0.4 seeds out of 10 seeds per tray) germinating in the vermiculite treatment than in the control (9.6 ± 0.4), salt (9.2 ± 0.4) or PEG (8.9 ± 0.4) treatments. There were no differences between the sites (*F* = 0.85; *P* = 0.4075), populations (*F* = 1.27; *P* = 0.1022) or maternal families (*F* = 0), nor was there any interaction between the site and the treatment (*F* = 0.30; *P* = 0.822) or between the population and the treatment (*Z* = 0.29; *P* = 0.378).

### Among-family and between-site variation

**Fig. 1:**
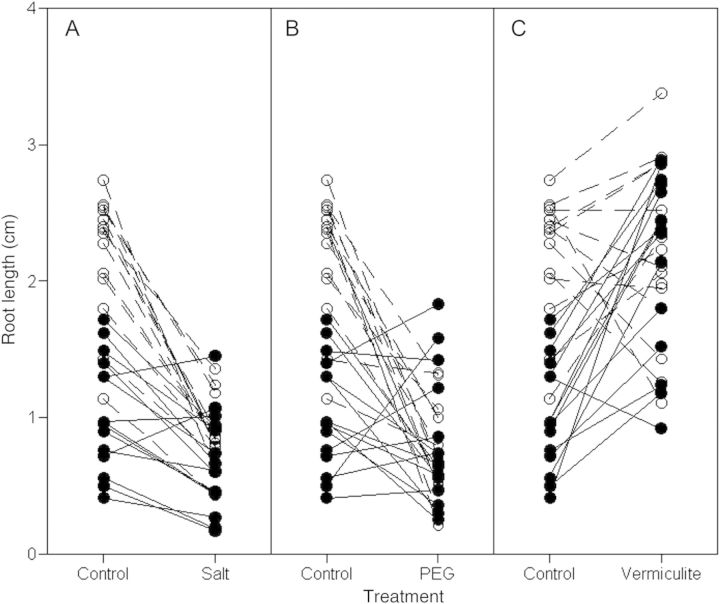
**Effects of salt and low osmotic potentials on seedling root length during germination.** *Dipsacus fullonum* seeds collected from old fields and roadsides were placed in (A) high-salinity (NaCl solution with a water potential of −0.5 MPa), (B) PEG (PEG 8000 solution with a water potential of −0.5 MPa), (C) vermiculite (mixed with distilled water to a water potential of −0.5 MPa) and control (distilled water) solutions. After 10 days the root length of each seedling was measured. Linear connections represent the averages from each maternal family. Dashed lines and open circles represent families from old field populations; solid lines and closed circles represent families from roadside populations.

Root length was significantly greater in vermiculite (2.26 ± 0.05 cm) than in distilled water controls (1.64 ± 0.04 cm) (*t* = −3.16; *P* = 0.008). Root length was significantly reduced in the salt (0.79 ± 0.02 cm) (*t* = 5.24; *P* < 0.001) and PEG (0.82 ± 0.03 cm) (*t* = 5.27; *P* < 0.001) treatments. As in previous studies (Beaton and Dudley 2004, 2007), although root length was affected by the treatments, the treatment effects depended on the site of origin (treatment × site *F* = 4.90; *P* < 0.019) and varied among families from the same population (family × treatment *Z* = 5.91; *P* < 0.001) but not among populations (Fig. 1).

In the control (distilled water) treatment, old field seedlings had longer roots than roadside seedlings (*t* = 4.48; *P* = 0.0008) (Fig. 1), while in the salt treatment, root length did not differ between old field and roadside seedlings (*t* = 1.26; *P* = 0.231). Salt tolerance (the family mean root length in salt/the family mean root length in distilled water) was greater in roadside families (*F* = 5.46; *P* = 0.027). In the PEG treatment, root length was significantly greater in roadside than in old field seedlings, and PEG tolerance was greater in roadside families than old field families (*F* = 7.67; *P* = 0.010). In the vermiculite treatment, root length did not differ significantly between roadside seedlings and old field seedlings (*t* = 0.12; *P* = 0.110), but vermiculite tolerance was greater in the roadside families (*F* = 13.46; *P* < 0.001).

### Genetic correlations

Genetic correlations between root lengths in the three low-osmotic-potential treatments (i.e. salt, PEG, vermiculite) were low in both the old field and roadside populations (Table 1) with the exception of the relatively high negative genetic correlation between the root length in vermiculite and the root length in PEG in the old field families. Root lengths in PEG and vermiculite were likewise not strongly correlated with root lengths in the control, with the exception of the rather strong negative genetic correlation between the root length in the control and the root length in PEG in the old field families (Table 1). In contrast, there were strong positive genetic correlations between root length in the control treatment and root length in the salt treatment in both roadside and old field families (Table 1).

**Table 1:** The genetic correlations (*r*_g_) between root lengths (log transformed prior to analysis) of seedlings germinated in distilled water (control), high salinity, PEG and vermiculite for *D. fullonum* seeds collected from old field and roadside locations.

	Old field and roadside	Old field	Roadside
Vermiculite × PEG	−0.35*	−0.49	−0.26
Vermiculite × salt	−0.20	0.14	−0.25
Salt × PEG	−0.01	−0.13	0.05
Control × vermiculite	0.17	0.21	0.32
Control × salt	0.60***	0.64*	0.60*
Control × PEG	−0.11	−0.44	−0.01

*P* values determined from the family mean correlations. *P < 0.05; ***P < 0.001.

### Correlations

**Fig. 2:**
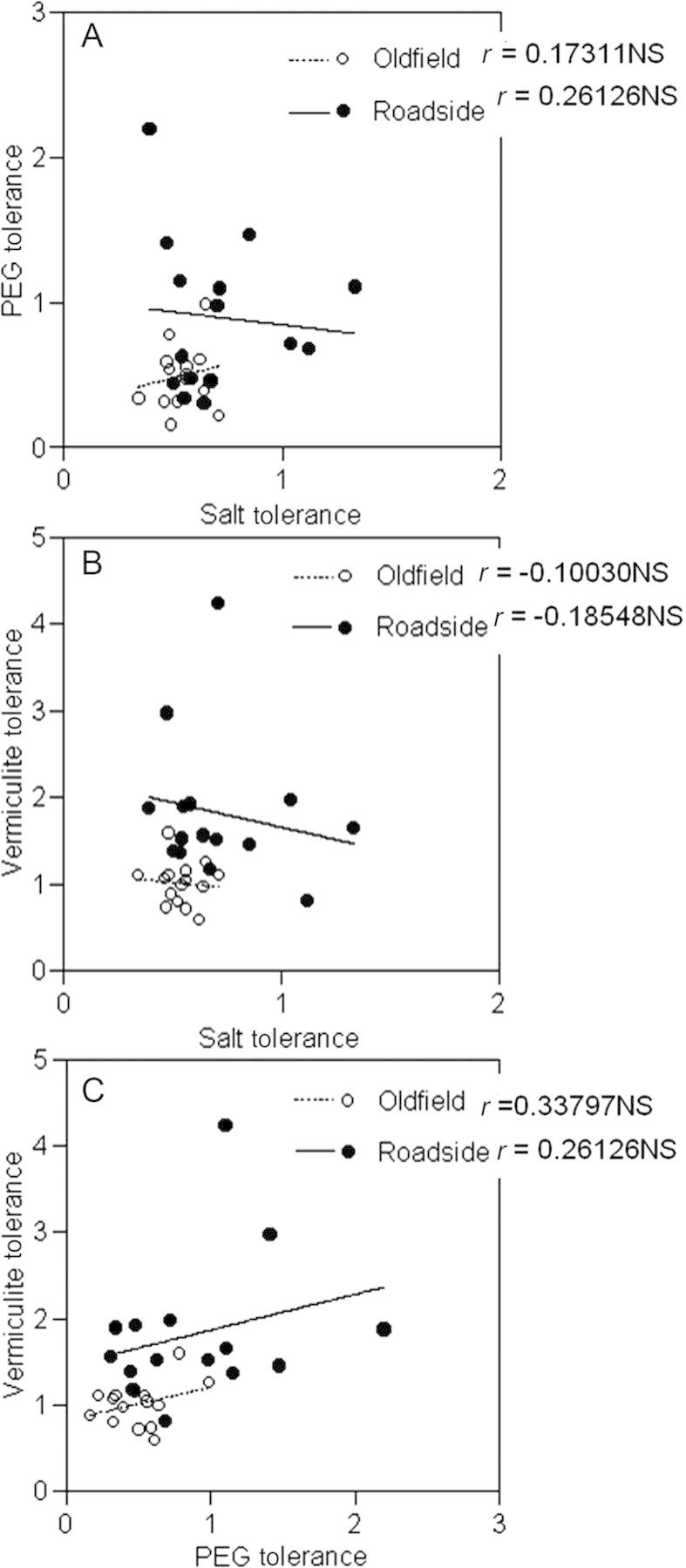
**The correlations (*r*) of the average PEG tolerance and salt tolerance (A), vermiculite tolerance and salt tolerance (B), and PEG tolerance and vermiculite tolerance (C) of maternal families collected from old fields and roadsides.** *Dipsacus fullonum* seeds collected from old fields (open circles) and roadsides (closed circles) were placed in high-salinity (NaCl solution with a water potential of −0.5 MPa), PEG (PEG 8000 solution with a water potential of −0.5 MPa), vermiculite (mixed with distilled water to a water potential of −0.5 MPa) and control (distilled water) solutions. After 10 days the root length of each seedling was measured. Tolerance to each stressor was calculated by dividing the average root length in the salt, PEG or vermiculite treatment by the average root length in the control. NS, not significant.

The roadside families demonstrated a greater range of values for tolerance to growth in low water potentials than the old field families. Several examples of tolerance values >1 indicate enhanced growth in a stress treatment compared with distilled water. However, high tolerance of one stress did not predict high tolerance of the other. Neither salt tolerance and PEG tolerance (Fig. 2A) nor salt tolerance and vermiculite tolerance (Fig. 2B) were significantly correlated in either roadside or old field maternal families, indicating that salt tolerance was not related to drought. Vermiculite tolerance and PEG tolerance were also not significantly correlated (Fig. 2C), although there was a weak positive non-significant relationship. Salt tolerance as indicated by cotyledon emergence was significantly correlated with salt tolerance as indicated by root length in both the old field (*r* = 0.567; *P* = 0.0275) and the roadside (*r* = 0.698; *P* = 0.0115), but was not correlated with PEG tolerance as indicated by root length (old field *r* = 0.289, *P* = 0.297; roadside *r* = 0.246, *P* = 0.440) or vermiculite tolerance as indicated by root length (old field *r* = −0.173, *P* = 0.538; roadside *r* = −0.2438, *P* = 0.4451).

### Cotyledon emergence

**Fig. 3:**
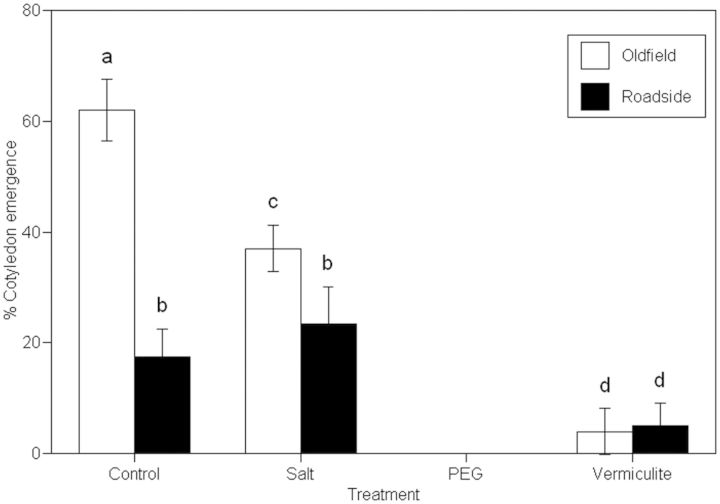
**Effect of high salinity and low osmotic potentials on cotyledon emergence.** The percentage of germinating *D. fullonum* seeds collected from old fields and roadsides and placed in high-salinity (NaCl solution with a water potential of −0.5 MPa), vermiculite (mixed with distilled water to a water potential of −0.5 MPa), PEG (PEG 8000 solution with a water potential of −0.5 MPa) and control (distilled water) treatments to have their cotyledons emerged from the tests after 10 days.

Old field families had more seeds with emerged cotyledons than roadside families (*F* = 22.73; *P* < 0.001; Fig. 3), particularly in the control treatment. The old field families responded in a significantly different manner to the treatments than the roadside families (*F* = 12.66; *P* < 0.001). While the old field families showed a sharp reduction in cotyledon emergence in response to the salt treatment, there was no significant difference in the response of the roadside families (Fig. 3). In response to low water potentials, plants are known to increase their relative allocation to roots at the cost of their investment in aboveground growth. In accordance with this, there was very low cotyledon emergence in both the PEG and the vermiculite treatments for both the old field and the roadside maternal families.

## Discussion

In this study, we took a quantitative genetics approach to examining the relationship between salt and drought tolerance. As in previous studies, maternal families from roadside populations had lower root growth in the control treatment than maternal families from old field populations (Beaton and Dudley 2004, 2007). Tolerance to particular environmental stresses is often accompanied by slower growth in a more benign environment (Chapin *et al*. 1993; Eränen 2008; Huttunen *et al*. 2010). For this reason, tolerance (i.e. the ability to maintain growth when exposed to a particular stress) cannot be assessed except by comparing the growth of genotypes in more than one environment (Simms 2000). The low growth rates of the roadside families in the control treatment may therefore be the cost of adaptation to the various abiotic stresses of the roadside environment.

Exposure to highly saline soils or solutions causes plants to experience both osmotic stress and toxicity from high Na^+^ levels. Although the stress responses to salt and drought have been shown to be intricately related (Seki *et al.* 2002), tolerance during germination to one of these stresses did not confer tolerance to the second in *D. fullonum* (Fig. 2). Although roadside families were better at tolerating low water potentials, imposed by vermiculite, PEG and high salinity, individual roadside families that possessed traits for salt-enhanced growth did not possess traits for drought tolerance.

In a previous study, Beaton and Dudley (2007) suggested that roadside populations of *D. fullonum* possessed two distinct salt tolerance strategies. Some families accumulated high levels of osmotica in the seeds to create low internal water potentials, which allowed the seed to imbibe water even in the highly saline springtime soils of the roadside environment. Other families possessed a trait for salt-enhanced growth that was not reliant on seed composition. We hypothesized that the trait for drought tolerance conferred by the composition of the seed was different from the trait for salt-enhanced growth. The results from the current study provide further evidence for a diversity of strategies to cope with salinity stress. The families that displayed salt-enhanced growth were not those that exhibited the strongest ability to tolerate drought stress.

The distinction between the salt tolerance and drought tolerance was also supported by how these stresses affected the allocation of resources between above- and belowground growth. The percentage of old field seedlings with emerged cotyledons in the salt treatment was lower than in the control treatment, but higher than in either of the low-water-potential treatments. In the vermiculite treatment, root length was significantly longer than in either the control or the salt treatment. Although this is likely to be a combination of low water potential and a thigmomorphogenic response related to the resistance of the growing medium (as discussed below), it indicates that seedlings from the roadside and old field sites were responding differently to the two treatments. In response to low water potential, a greater proportion of available resources was dedicated to root growth at the expense of shoot growth, while in response to high salinity, resources were not diverted exclusively to belowground growth. The divergence of resources from shoots to roots is a common response to drought stress (Sharp *et al*. 1988; Lenzi *et al*. 1995). Investing more resources in root growth increases the likelihood that the plant will gain increased access to water. This is an especially crucial response for seedlings because they inhabit the surface soil, which is the first to dry out when water becomes limiting. The deeper that roots are able to penetrate, the greater the likelihood for survival of the seedling. Investing in root growth at the expense of shoot growth in response to salinity stress would not provide the same benefits. First, the plant is not likely to encounter a source of non-saline water by investing more in root growth, and second, since many plants deposit excess Na^+^ in their leaves, shoot growth can benefit plants in high salinity. An increase in root surface area may expose the plants to higher Na^+^ levels. Additionally, tolerating high Na^+^ levels may require a large resource investment that requires actively photosynthesizing tissues.

The germinating seedlings responded differently to the vermiculite and PEG treatments. Although both impose low water potentials, the PEG solution has the additional impact of causing hypoxia (Mexal *et al*. 1975). As oxygen demand increases under low water potentials (Verslues *et al*. 1998), the reduced oxygen availability would be additionally harmful to the germinating seeds. It is notable that the roadside families exhibited an increased ability to tolerate the PEG solutions when compared with the old field families. This result indicates that, in addition to being better able to tolerate low water potentials, the roadside families were also better able to tolerate low-oxygen conditions. Roadside populations in the study region are often found in extremely wet areas with poorly drained clay soil (L. L. Beaton, pers. observ.). This type of environment is very likely to impose hypoxic conditions. In contrast, in the old field, *D. fullonum* populations are often found in comparatively dry, well-drained environments.

A second difference between the vermiculite and PEG treatments is the texture of the media. It has been suggested that the morphology of plants growing in liquid media will be different from that when they are grown in solid media. In the vermiculite, the seedlings produce roots that are much longer and thinner than in solution. However, this is not likely to be a stress response, since the vermiculite was not firmly packed. Therefore, we expect that many of the differences in root length reflect that the roadside and the old field families differed in their ability to tolerate low water potentials. Several old field families had shorter roots in the vermiculite than in the control treatment. In contrast, all but one roadside family grew longer roots in the vermiculite than the control treatments. There was a significant level of variation in the tolerance of the old field families to low water potentials. Drought stress occurs as a result of freezing temperatures as well as drought and high salinity (Verslues *et al*. 2006). While salinity stress occurs predictably every year in the roadside, freezing and drought occur randomly, depending on climatic conditions. Unlike old field soil, which is generally a very rich topsoil, the small soil particles of the heavy clay soil of the roadside tightly bind water, decreasing the amount of water available to plants (Rosenthal *et al*. 2005). This harsh environment could act as an extremely effective selection pressure. Despite the random nature of the occurrence of droughts, roadside genotypes that are not highly tolerant of low water potentials would have very low fitness during any drought event. As long as the costs associated with drought tolerance are low, the more mesic soil of the old field should not select as strongly for tolerance of low water potentials, leading to the retention of variation.

It has often been suggested that tolerance to different environmental stresses is obtained at the cost of growth in the absence of the stress. Traits that are not associated with a cost are more likely to evolve. Average root lengths in the salt treatment of both old field and roadside families were positively correlated with root lengths in the control treatment, indicating that salt tolerance is more likely to evolve in *D. fullonum* than either PEG or vermiculite tolerance. In contrast, among old field families, root length in the PEG treatment was negatively correlated with root length in the control treatment, indicating a cost of tolerance to the stresses (i.e. low osmotic potential and hypoxia). Hence, drought tolerance as revealed by PEG is not as likely to have evolved among old field families. The lack of a negative correlation in root length in the salt in relation to growth in the PEG and vermiculite suggests that although salt tolerance does not confer tolerance towards drought or hypoxia, there is also no cost associated with adaptation to high salinity in terms of the ability to adapt to low osmotic potentials and hypoxia. Interestingly, a recent study revealed the conference of drought stress by high salinity among three *Atriplex* species (Glenn *et al*. 2012).

## Conclusions and forward look

Despite the high availability of nitrogen, the roadside environment is stressful and subjects populations to many different selection pressures. The lack of overlap in tolerance trait for salinity and drought between maternal families may indicate that populations could not respond simultaneously to the two selection pressures. The contrasting responses of genotypes to different selection pressures suggest that the absence of local adaptation found in previous studies (Beaton and Dudley 2010) may be the result of the complexity of selection pressures operating in this novel habitat. Genetic correlations appear not to constrain evolution, but also not to enhance the response to multiple selection pressures. Because *D. fullonum* is able to adapt rapidly to many different environmental conditions, it is more likely that this species will be able to spread rapidly into new habitats from the roadside and pose a significant threat as an invasive plant. Future studies examining the role of maternal environmental effects in early stress tolerance traits may help to explore the establishment success of this species in the novel roadside environment.

## Sources of funding

The work was funded by a grant from the Natural Science and Engineering Research Council of Canada to S.A.D.

## Contributions by the authors

L.L.B. designed and conducted the experiment, analysed the data, produced the figures and tables, and wrote the paper. S.A.D. provided advice/instructions on statistical analyses, figure design and significant editorial comments on the manuscript.

## Conflict of interest statement

None declared.
